# Marine Algae Bioactives: Isolation, Characterization, and Potential Application

**DOI:** 10.3390/foods13111736

**Published:** 2024-06-01

**Authors:** Ivana Generalić Mekinić, Vida Šimat

**Affiliations:** 1 Department of Food Technology and Biotechnology, Faculty of Chemistry and Technology, University of Split, 21000 Split, Croatia; 2 Department of Marine Studies, University of Split, R. Boškovića 37, 21000 Split, Croatia; vida@more.unist.hr

This Special Issue (SI) of *Foods*, entitled “*Marine Algae Bioactives: Isolation*, *Characterization*, *and Potential Application*”, was focused on algal organisms, both microalgae and macroalgae, which have recently been recognized as new, cost-effective, and valuable sources of health-promoting nutrients and bioactive compounds with a full spectrum of activities and beneficial effects on health. The focus of this SI was also on the methods and technologies used in the isolation, purification, and identification of these bioactives, as well as their application in various fields and industries.

In this context, this SI consists of six original research papers [1,2,3,4,5,6] and two review papers [7,8] that address recent advances and current knowledge in the proposed field.

The isolation methods and the content of proteins, carbohydrates, minerals, lipids, and neutral fats from the four microalgae *Chlorella vulgaris* Beijer, *Arthrospira platensis* Gomont, *Arthrospira platensis* (Nordst.) Geitl., and *Dunaliella salina* Teod. were investigated in the study by Babich et al. [1]. In addition to the detection of high amounts of valuable nutrients in the algal samples examined, the authors reported that the composition of the nutrient medium influenced the lipid content and chemical profile of the algae.

Circuncisão et al. [2] investigated the extraction protocols and steps to modulate the composition of food-grade extracts/fractions from the brown alga *Fucus vesiculosus*, which will enable their use as tailored functional ingredients in various foods. The compounds of primary interest were phenolic compounds (phlorotannins), xanthophyll pigments (fucoxanthin), and carbohydrates (alginates, fucoidans, and laminarans). This study emphasizes that the extraction protocol, in particular the solvent used, significantly influences the yield in the extraction of the compounds and that water at room temperature can be an efficient, cost-effective, and environmentally friendly solvent for the production of high-quality algal extracts.

The use of novel technologies, in this case supercritical fluid extraction (SFE), to isolate bioactive algal constituents was investigated by Martí-Quijal et al. [3]. The authors reported that the use of SFE to produce *Spirulina* extracts can lead to extracts with a higher content of valuable components such as fatty acids, phenolic compounds, minerals, pigments, etc. However, they also pointed out that special attention should be paid to the extraction yield of potentially harmful components, such as heavy metals.

Ferreira-Anta et al. [4], on the other hand, tested the effects of different temperatures (from 160 to 220 °C) in pressurized hot water extraction on the yield and profile of extracted bioactive components (proteins, fucoidan, phloroglucinol, carbohydrates, sulfates, etc.) from the edible brown alga *Undaria pinnatifida*. The authors also tested the antioxidant activity of the extracts and their possibility of encapsulation with mannitol, which may additionally enable their application in the food industry.

The use of two algae, the brown alga *Himanthalia elongata* and the cyanobacteria *Spirulina*, in the food industry was reported in a paper by Oliveira et al. [5]. The aim of the study was based on the idea of incorporating a mixture of algae into whole-wheat pasta, which may improve its nutritional and bioactive properties. The authors investigated the fat, protein, fiber, and ash content as well as the antioxidant composition of pasta with and without algae addition and, finally, the cooking effect on pasta properties. They concluded that algal addition can be an effective strategy to improve the nutritional and bioactive parameters of pasta.

In addition to the direct addition of algae to various foods, scientists are now also researching the incorporation of bioactive algal extracts into functional food packaging, which can extend the shelf life of foods and ensure greater product safety and quality. In this Special Issue, Čagalj et al. [6] published a paper in which they investigated seasonal variations in the composition and antioxidant activity of the brown alga *Padina pavonica*. They used microwave-assisted extraction to prepare the extracts. The extract with the highest phenolic content and antioxidant activity (algal material harvested in June) was used in combination with chitosan for the development of a bioactive polylactic acid film that showed anti-fogging and antioxidant effects, suggesting that extracts from *P. pavonica* could be used for the development of active food packaging solutions [6].

Finally, this SI also contains two revisions. The first, by Generalić Mekinić et al. [7], deals with the presence, extraction, and detection of carotenoids, which are among the most studied bioactive metabolites of algae. The review provides an overview of the latest findings on the total content of carotenoids and/or the presence and quantity of individual compounds detected in 12 microalgae and 90 macroalgae species.

In the second review paper, Lomartire and Gonçalves [8] gave an overview of macroalgal polysaccharides (agar, carrageenan, and alginate), their structure, and their properties, which vary depending on different parameters such as species, algal life cycle, abiotic and biotic factors, etc. The authors focused on the methods and techniques commonly used for the extraction of polysaccharides from algae, both conventional and novel, as well as their therapeutic effects and application in various foods and dietary supplements.

We thank all the authors who contributed to this Special Issue with their scientific knowledge and experience, as well as all the reviewers who evaluated the published manuscripts and significantly improved them with their comments and suggestions. Finally, we thank MDPI and *Foods* for all their help and support, which contributed to the success of the edition.
